# Lymphoid to Myeloid Cell Trans-Differentiation Is Determined by C/EBPβ Structure and Post-Translational Modifications

**DOI:** 10.1371/journal.pone.0065169

**Published:** 2013-06-05

**Authors:** Bilyana Stoilova, Elisabeth Kowenz-Leutz, Marina Scheller, Achim Leutz

**Affiliations:** 1 Max-Delbrueck-Center for Molecular Medicine, Berlin, Germany; 2 Berlin-Brandenburg Center for Regenerative Therapies, Berlin, Germany; 3 Humboldt-University of Berlin, Institute of Biology, Berlin, Germany; Ludwig-Maximilians-Universität München, Germany

## Abstract

The transcription factor C/EBPβ controls differentiation, proliferation, and functionality of many cell types, including innate immune cells. A detailed molecular understanding of how C/EBPβ directs alternative cell fates remains largely elusive. A multitude of signal-dependent post-translational modifications (PTMs) differentially affect the protean C/EBPβ functions. In this study we apply an assay that converts primary mouse B lymphoid progenitors into myeloid cells in order to answer the question how C/EBPβ regulates (trans-) differentiation and determines myeloid cell fate. We found that structural alterations and various C/EBPβ PTMs determine the outcome of trans-differentiation of lymphoid into myeloid cells, including different types of monocytes/macrophages, dendritic cells, and granulocytes. The ability of C/EBPβ to recruit chromatin remodeling complexes is required for the granulocytic trans-differentiation outcome. These novel findings reveal that PTMs and structural plasticity of C/EBPβ are adaptable modular properties that integrate and rewire epigenetic functions to direct differentiation to diverse innate immune system cells, which are crucial for the organism survival.

## Introduction

Understanding the molecular attributes and post-transcriptional regulation of transcription factors in cell fate determination remains a challenging task in molecular genetics and developmental biology. Ectopic expression of some key transcription factors can perturb cellular differentiation programs and install new ones, such as during lymphoid to myeloid reprogramming or trans-differentiation induced by CCAAT enhancer binding proteins (C/EBPs) [1], [2]. Trans-differentiation experiments may help to determine plasticity of cell differentiation and how lineage decisions are accomplished and epigenetically fixed, providing important information for future regenerative medicine.

C/EBPs are gene regulators involved in many cell differentiation and growth control processes in different cell types, including cells from the hematopoietic system [3]. C/EBPβ trans-differentiates B lymphoid cells into inflammatory macrophages, activates eosinophil genes in hematopoietic progenitors, acts as a pioneering factor during dendritic cell (DC) specification and is involved in emergency granulopoiesis [4], [5], [6], [7], [8], [9], [10]. C/EBPβ orchestrates cell type specification in combination with other transcription factors and co-factors: C/EBPβ together with c-Myb activates myeloid genes in fibroblasts, together with PU.1 evokes macrophage differentiation, and together with TAL1 and FLI1 binds to and establishes early priming of hematopoietic lineage genes [11], [12], [13].

Structurally, C/EBPs contain N-terminal transactivation domains (TAD), central regulatory domains (RD) and C-terminal DNA-binding and leucine zipper dimerization domains (bZip). The TAD and RD display modular designs with several highly conserved regions (CRs) that are separated by polymorphic low complexity regions (LCRs) [14], [15]. C/EBPβ is extensively modified by post-translational modifications (PTMs), including lysine acetylation, mono-, di-, tri-methylation, arginine mono- and di-methylation, in addition to serine, threonine, and tyrosine phosphorylation [3], [15], [16], [17], [18]. Moreover, alternative translation initiation generates N-terminally truncated isoforms which further multiplies C/EBPβ diversity [18], [19]. Natural N-terminal, or experimental intra-molecular deletions or PTM site mutations suggest modular, context specific functions of C/EBPβ. The emerging view is that multi-site modifications of C/EBPβ integrate extracellular signals to alter scaffolding functions for recruitment of chromatin modulating complexes and the basic transcription machinery [15], [17], [20], [21].

To answer the emerging question about the importance of C/EBPβ structure and PTMs for determination of cell fate, here we used an assay for trans-differentiation of primary B lymphoid into myeloid cells [4], [10]. We identified the essential requirement of a core trans-activating region of C/EBPβ that was previously shown to interact in a regulated fashion with several transcription factors and co-factors. Distinct C/EBPβ PTM site or CR mutations variegate reprogramming outcomes to yield cellular phenotypes that correspond to at least four different myeloid cell types. Interestingly, the granulocytic outcome depends on the capacity of C/EBPβ to recruit chromatin remodelers. Our data demonstrate that a multitude of PTMs in connection with structural plasticity are pivotal for the fine-tuning of the epigenetic C/EBPβ functions to determine cell fate in the innate immune system.

## Results and Discussion

### The B cell to Myeloid Reprogramming Potential Resides in the C/EBPβ TAD

**Figure 1:**
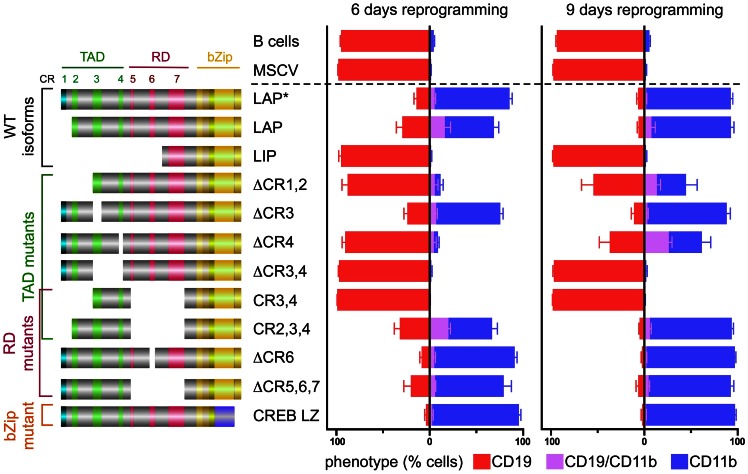
Structural requirements for B cell to myeloid reprogramming potential of C/EBPβ. Schematic representation of the different C/EBPβ constructs (left) indicating the conserved regions (CRs) in the transactivation domain (TAD; CR1,2,3,4; green, turquoise), regulatory domain (RD; CR5,6,7; red), bZip domain (yellow), and the low complexity regions (LCRs, grey). Expression of lineage specific markers: B cell CD19 (red), myeloid CD11b (blue), or double positive (magenta) at 6 (middle panel) or 9 dpi (right panel). Bar graph shows percentage of GFP^+^ gated (virus infected) cell population; B cells - control uninfected GFP^–^ B cell progenitors. Results represent mean ± SEM from at least two experiments.

To identify C/EBPβ structures involved in lympho-myeloid trans-differentiation, primary B cell progenitors were purified from wild type (WT) mouse bone marrow (. S1A) and retrovirally infected with C/EBPβ constructs, including the three C/EBPβ isoforms (LAP*, LAP, and LIP), as well as various CR recombinants (Fig. 1, left panel). Infected cells were cultured under conditions that support both B cell and myeloid cell development [10] and surface marker expression alterations were analyzed by flow cytometry (FACS) at 6 and 9 days post-infection (dpi) to monitor reprogramming kinetics (Fig. 1 and S2A). Both the LAP* and LAP C/EBPβ isoforms up-regulated the myeloid surface marker CD11b and down-regulated the B cell marker CD19 at 6 and 9 dpi, indicating the gradual loss of the B cell phenotype and completion of lympho-myeloid trans-differentiation. In contrast, no significant change in the B cell phenotype was observed in cells infected with the LIP C/EBPβ isoform, similarly to cells infected with MSCV vector or uninfected controls (Fig. 1 and S2A).

LAP* and LAP isoforms are distinguished by CR1, which determines SWI/SNF chromatin remodeling complex recruitment and differential regulation of gene subsets [20], [22], [23]. Omission of CR1, as in the LAP isoform or in the CR2,3,4 mutant, significantly decreased the kinetics of both acquisition of myeloid and annulation of B cell features (Fig. 1, S2A and Table S1). Deletion of CR1,2 or CR4 strongly compromised but did not entirely abolish reprogramming, whereas removal of CR3 did not affect trans-differentiation. Deletion of CR3,4 (ΔCR3,4) entirely abrogated both activation of CD11b and repression of CD19, however CR3,4 in combination with the bZIP was not sufficient for reprogramming but required CR2 (CR2,3,4 in Fig. 1 and S2A). The core trans-activating region of C/EBPβ CR2,3,4 was previously shown to interact in a regulated fashion with several transcription factors and co-factors, including CBP/p300, CARM1/PRMT4, G9a, TBP/TFIIB, Mediator, and several other chromatin regulatory complex components [20], [21], [22], [24], [25], [26], [27], [28]. The LIP isoform, which lacks transactivation potential and acts as a dominant negative inhibitor, not only failed to induce myeloid conversion but also failed to down-regulate B cell marker expression. Thus, activation of the myeloid program and shutting down the B cell program both reside in the C/EBPβ TAD. As suppression of B cell fate involves removal of Pax5 [10], one may therefore infer that inhibition of Pax5 occurs through C/EBP mediated activation of a Pax5 inhibitor, co-repressor, inhibitory RNA, or proteolysis.

In many cell types C/EBPβ is auto-repressed and becomes activated by receptor tyrosine kinase ras/MAPK signaling, resulting in acquisition of several C/EBPβ PTMs and alterations of protein interactions [14], [16], [21], [25], [29]. In fibroblasts and erythroblastoid cells deletion of the repressive RD (ΔCR5,6,7) enhanced myeloid gene activation by C/EBPβ, whereas removal of CR6 (ΔCR6) represented a dominant-negative mutant [14]. Surprisingly, both RD mutants ΔCR5,6,7 and ΔCR6 displayed trans-differentiation potential similar to LAP*, suggesting that regulation of C/EBPβ in B cells may differ from other cell types. The kinetics of myeloid trans-differentiation by a leucine-zipper exchange mutant (CREB LZ) was found to be similar to WT, suggesting that i) C/EBPβ homodimers are able to reprogram B cells, ii) the major trans-differentiation function of C/EBPβ resides in the TAD, and iii) both the bZip and the RD structures play minor roles in lineage conversion. Notably, the reprogrammed myeloid cells showed immunoglobulin gene rearrangement, confirming their B cell origin (1B).

To exclude auto-regulatory activation of endogenous C/EBPβ during lineage conversion C/EBPβ deficient B cell progenitors were tested. No differences between C/EBPβ isoform or mutant trans-differentiation capacity were observed between primary WT and *C/EBPβ^−/−^* B cell progenitors (Fig. S2B compared to Fig. 1). Likewise, no difference in the reprogramming capacity of C/EBPα p42 was detected when WT and *C/EBPβ* deficient B cells were compared (Fig. S2C). Furthermore, the truncated C/EBPα p30 isoform, which lacks the C/EBPα TAD (equivalent to C/EBPβ CR2,3,4 TAD) failed to reprogram WT B cells, suggesting that major reprogramming functions of both, C/EBPα and C/EBPβ, reside within their TADs. Therefore, C/EBPα- and C/EBPβ-mediated reprogramming are direct effects of the ectopically expressed transcription factors.

### Differential Regulation of Key Myeloid Genes by C/EBPβ WT and Mutants

**Figure 2:**
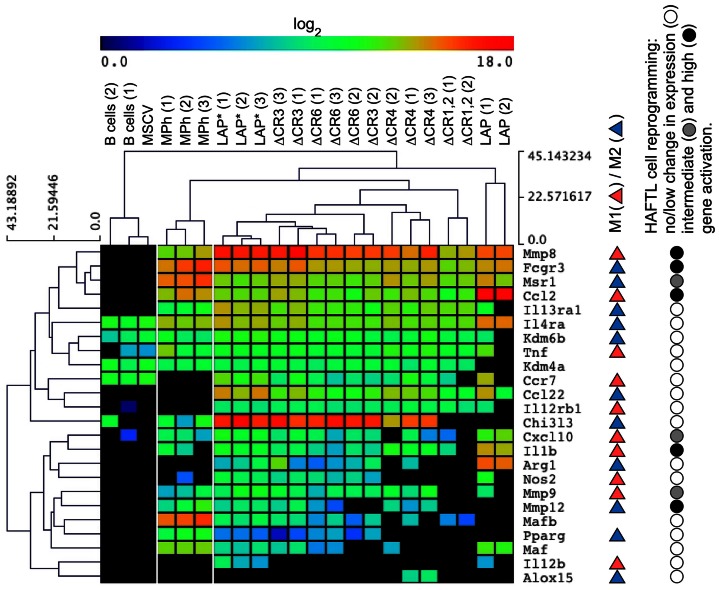
C/EBPβ WT and mutants differentially regulate key myeloid genes. RNA counts for pro-inflammatory M1, anti-inflammatory M2 and other key monocyte/macrophage genes evaluated on CD11b^+^ reprogrammed *C/EBPβ* ^−/−^ B cell progenitors. Data were calculated as log_2_ and subjected to hierarchical clustering. Results represent expression profiles from three independent experiments. On the right, comparison to data obtained from reprogramming of pre-B cell line by C/EBPα is presented (Bussmann et al., 2009). MPh - WT bone marrow-derived macrophages.

To further analyze how the C/EBPβ structure contributes to myeloid gene expression, several pro-inflammatory M1, anti-inflammatory M2 genes, and key regulators of macrophage differentiation were examined by NanoString technology. RNA expression analyses of *C/EBPβ^−/−^* B cell progenitors reprogrammed by WT and mutant C/EBPβ showed that many M1 genes and M2 genes became up-regulated during trans-differentiation (Fig. 2). Hierarchical gene clustering indicated no prevalence in M1 or M2 gene expression in reprogrammed cells and an overlap but also differences between C/EBPβ and C/EBPα activated genes [4]. The C/EBPβ isoform LAP* and the deletion mutants ΔCR3 and ΔCR6 activated the majority of analyzed genes. Other constructs, including LAP, ΔCR1,2 and ΔCR4, showed lower or lacked trans-activation potential for several M1 and M2 genes. Both, the LAP C/EBPβ isoform and the ΔCR1,2 mutant failed to up-regulate several macrophage polarization genes, including *Mmp12*, *Pparg*, and *Chi3l3*, suggesting that SWI/SNF recruitment through CR1 is a prerequisite for their activation [20], [22]. Several other genes (*Cxcl10*, *Arg1*, *Maf*) were up-regulated by LAP but not by ΔCR1,2, suggesting that these genes require CR2 functions that are distinct from SWI/SNF recruitment. Finally, some genes (*Il1b*, *Cxcl10*, *Ccl2*, *Arg1*, *Il4ra*, *Maf*) were more strongly activated by LAP than LAP*, in agreement with isoform-specific gene regulatory functions [30]. On the other hand, LAP* and several C/EBPβ deletion mutants, but not LAP, activated the expression of *Mafb*, whereas LAP was the strongest activator of the *Maf* gene. In macrophage gene regulatory circuitry, the lysine-specific demethylase 6B *Kdm6b* (*Jmjd3*) is important for M2, but not for M1 polarization [31], [32]. Interestingly, LAP and ΔCR1,2, which showed lower activation of *Kdm6b* expression (3–6-fold) as compared to LAP*, both failed to up-regulate *Chi3l3*, and ΔCR1,2 reprogrammed cells did also not express *Arg1* (Fig. 2). Hence, many myeloid genes displayed designated C/EBPβ CR-specific regulation, suggesting complex combinatorial, locus specific relevance of distinct C/EBPβ CRs in gene regulation.

### The C/EBPβ Structure Determines Alternative Trans-differentiation

Previously, it has been shown that C/EBPα or -β trans-differentiate B cell progenitors only into inflammatory macrophages, characterized as CD11b^+^ F4/80^+^ Gr-1^+^ CD62L (L-selectin)^+^ phenotype [10]. Phagocytosis assays performed with C/EBPβ reprogrammed CD11b^+^ cells, however, suggested cell heterogeneity (Fig. S3A). In conjunction with the kaleidoscopic myeloid gene regulation repertoire of C/EBPβ mutants (Fig. 2), this prompted us to explore the possibility of trans-differentiation into distinct cell types. To this end, CD11b^+^ cells were examined for expression of Gr-1/Ly-6C to distinguish between inflammatory (CD11b^+^ Gr-1/Ly-6C^+^) and resident type (CD11b^+^ Gr-1/Ly-6C^–^) monocytes/macrophages [33]. At 6 dpi the LAP* isoform generated two CD11b^+^ subpopulations, with predominance of CD11b^+^ Ly-6C^+^ cells and at 9 dpi the percentage of Ly-6C^+^ cells significantly decreased at the expense of Ly-6C^–^ cells (Table S2). No differences in the frequency of apoptotic cells was observed (Fig. S3B), suggesting that the reduction of Gr-1/Ly-6C^+^ cells was not caused by selective cell death. Interestingly, C/EBPβ constructs that lacked CR1 (LAP, CR2,3,4, ΔCR1,2) induced less Ly-6C^+^ cells, while others (ΔCR6) strongly induced Ly-6C^+^ cells at both 6 and 9 dpi (Table S2). These results suggested not only cell heterogeneity but also that the C/EBPβ structure might determine the myeloid phenotype.

**Figure 3:**
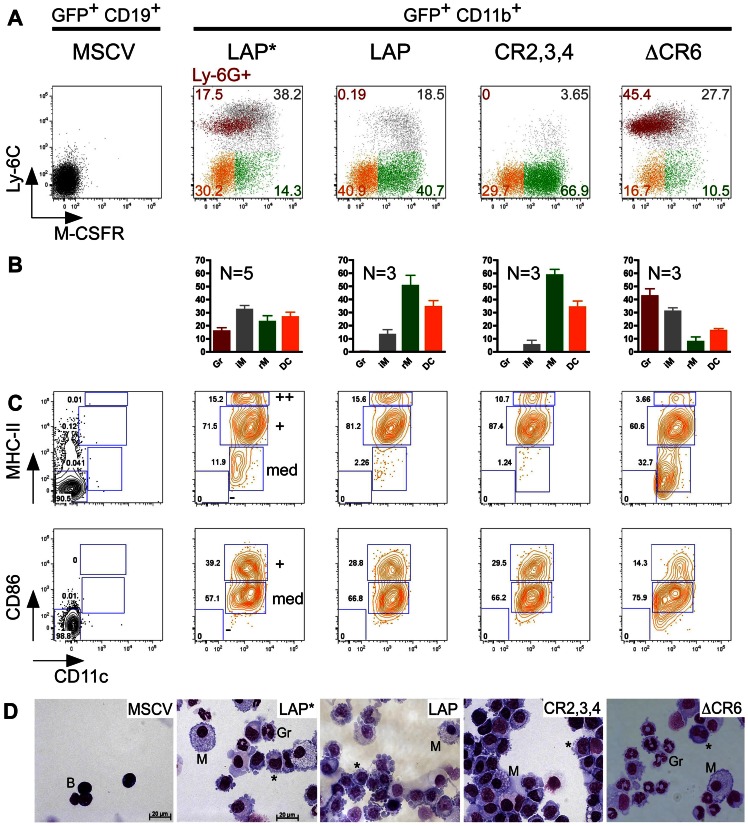
C/EBPβ structural mutants define distinct myeloid cell trans-differentiation outcomes. A. Representative FACS plots depicting the expression of myeloid cell markers Ly-6C, M-CSFR, and Ly-6G on 9 days trans-differentiated cells. FACS plots represent GFP^+^ CD11b^+^ cell populations, for MSCV control - GFP^+^ CD19^+^ cells. B. Distribution of the myeloid subpopulations among the reprogrammed GFP^+^ CD11b^+^ cells after staining as in A and presented as mean ± SEM. N - number of repetitions. Gr - neutrophil granulocytes, iM and rM - inflammatory and resident monocytes/macrophages, respectively, DC - dendritic cells. C. Expression of the DC markers CD11c, MHC-II and CD86 on the reprogrammed Ly-6C^–^ M-CSFR^–^ cells. Histograms represent GFP^+^ CD11b^+^ Ly-6C^–^ M-CSFR^–^ gated cells (color coded as the corresponding population on the Ly-6C/M-CSFR plot in A). “++”, “+”, “med” and “−” represent the expression levels of MHC-II and CD86 antigens. D. Cytospins of control MSCV infected CD19^+^ cells and CD11b^+^ cells reprogrammed by WT C/EBPβ or deletion mutants. B – B cells, M – macrophages, Gr – neutrophil granulocytes, * - monocytes/DCs.

Ly-6C/Gr-1 expression distinguishes inflammatory from resident monocytes/macrophages [33]. Lack of MCSF-R could serve to discriminate granulocytes from monocytes/macrophages, however, as MCSF-R is also a direct C/EBPβ target gene [34], Ly-6G was included as a neutrophil granulocytic surface marker [35]. Based on the expression of Ly-6C, MCSF-R, and Ly-6G, the C/EBPβ-LAP* reprogrammed CD11b^+^ cells consisted of four cell subpopulations: resident monocytes/macrophages (Ly-6C^–^ M-CSFR^+^), neutrophil granulocytes (Ly-6C^+^ Ly-6G^+^), and Ly-6C^–^ M-CSFR^–^ cells (Fig. 3A, B), in addition to the previously shown inflammatory monocytes/macrophages (Ly-6C^+^ Ly-6G^–^) [10]. The Ly-6C^–^ M-CSFR^–^ cells were further analyzed and classified as CD11c^+^ MHC-II^+/++^ CD86^+/med^, suggesting conventional dendritic (cDC) phenotype (Fig. 3C) [36]. The percentage of inflammatory monocyte/macrophages decreased between 6 and 9 dpi, whereas the percentage of resident monocytes/macrophages increased (Fig. S3C). This is most likely due to differentiation of inflammatory monocytes/macrophages into resident ones [33]. Cyto-morphological examination of the LAP*-reprogrammed cells confirmed FACS data and revealed the presence of cells with morphological characteristics of polymorphonuclear neutrophils, monocytes/DCs, and macrophages (Fig. 3D), whereas the MSCV control or C/EBPβ constructs incapable of inducing CD11b expression (such as LIP, ΔCR3,4, CR3,4) displayed B cell phenotype (Fig. 3). No granulocytic differentiation and only few inflammatory monocytes/macrophage were obtained by constructs lacking CR1, such as LAP, CR2,3,4 and ΔCR1,2 (Figure 3A, B, D and S3C). In contrast, deletion of CR6 led to an increase in the neutrophil granulocytic population (Fig. 3A, B, D). Interestingly, the augmented granulocytic differentiation correlated with decreased DC differentiation (Fig. 3A, B, C). Based on myeloid surface marker expression and cell morphology, we conclude that structural alterations in C/EBPβ pre-define the reprogramming outcomes into inflammatory and resident monocytes/macrophages, cDC-like cells, and granulocytes.

**Figure 4:**
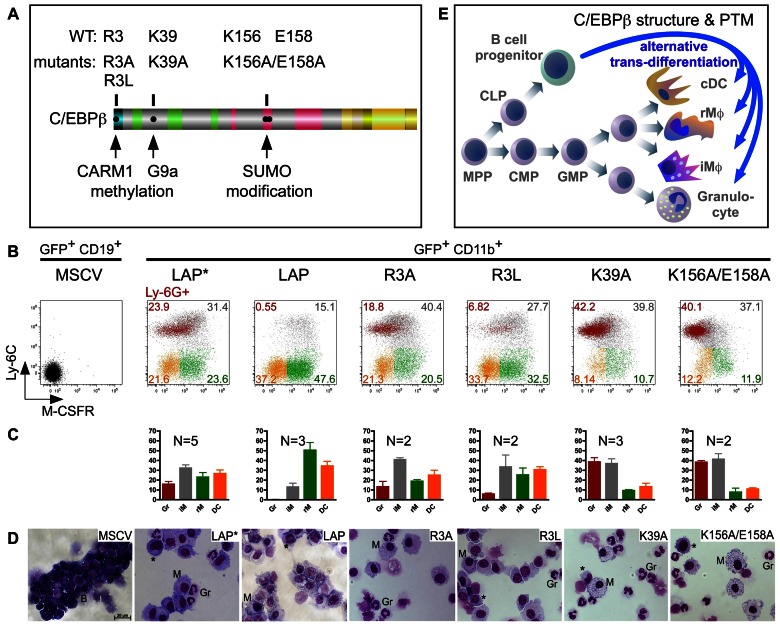
C/EBPβ PTM site mutations affect lympho-myeloid trans-differentiation. A. Schematic representation of C/EBPβ PTM sites and mutants tested in B-D. B. Expression of Ly-6C, M-CSFR and Ly-6G on the reprogrammed cells at 9 dpi. C. Distribution of the different myeloid populations among the reprogrammed GFP^+^ CD11b^+^ cells, stained as in B and presented as mean ± SEM. D. Cytospins of trans-differentiated sorted cells. Experiments were repeated two to three times and similar results were obtained. Gating strategies and abbreviations as in Fig. 3. E. Schematic representation of the normal hematopoiesis and lympho-myeloid reprogramming by C/EBPβ. MPP - multi potent progenitors, CLP - common lymphoid progenitor, CMP - common myeloid progenitor, GMP - granulocyte/macrophage progenitor, iMΦ and rMΦ - inflammatory and resident monocytes/macrophages.

Mechanistically, differences between LAP* and LAP have previously been attributed to differentially regulated SWI/SNF recruitment. LAP*-specific CR1 functions and the activity of the TAD have been shown to be negatively regulated by CARM1/PRMT4 and G9a methylation of R3 and K39, respectively [20], [22], [27]. Furthermore, CR1 was reported to control SUMOylation [30], thus integrating various signals to yield epigenetic consequences. Accordingly, we refined the trans-differentiation analysis using C/EBPβ point mutants that affect the above mentioned modification sites. As shown in Figure 4, amino acid substitution of the G9a K39 methylation sites or the UBC9 binding/SUMOylation/methylation sites K156A/E158A, enhanced granulocytic trans-differentiation, similar to ΔCR6 (Fig. 3). The LAP* R3L mutant, which mimics the R3 methylated state, abrogated SWI/SNF recruitment, and failed to induce the neutrophil elastase gene [20], strongly decreased granulocytic trans-differentiation, whereas the LAP* R3A mutant, which abrogates methylation, maintained granulocytic trans-differentiation (Fig. 4). Therefore, decoration of C/EBPβ with PTMs modifies its trans-differentiation capacity and, in agreement with other data [37], that recruitment of chromatin remodeling complexes through CR1 is required for granulocytic differentiation (Fig. 4E).

Advancing our understanding of the importance of transcription factor regulation and PTMs in lineage decisions is instrumental to elucidate normal development and aberrant epigenetic processes in connection with disease. Previous findings have suggested that chromatin regulatory factors and epigenetic state regulation are involved in hematopoietic cell decisions [37], [38]. Furthermore, it has been shown that interactions between C/EBPβ and the transcriptional and epigenetic machineries are controlled by C/EBPβ PTMs [15], [17], [18], [20], [21], [22], [25], [27] but their importance for directing differential myeloid cell differentiation is quite obscure. The B cell to myeloid lineage conversion now connects C/EBPβ PTMs to alternative cell fate instruction, raising the possibility that related mechanisms control regular myelopoiesis. Although we do not imply B cell to myeloid trans-differentiation as a frequent event, it recalls the evolutionary relationship between innate and acquired immunity [39], [40], [41], [42]. Moreover, lineage switching of B cell lymphoma to acute monoblastic leukemia or trans-differentiation of follicular lymphoma to histiocytic/DC sarcomas have been reported [43], [44] and bi-phenotypic lymphoma displayed functional dependency on high C/EBPβ expression [45], [46], [47]. These data suggest a role of lympho-myeloid plasticity in malignant transformation. It is evident that more detailed mechanistic insight in spatio-temporal modifications and co-factor recruitment requires advanced tools, such as generation of knock-in mouse mutants, determination of the PTM-dependent C/EBPβ interactome, PTM specific antibodies and genome wide comparison of C/EBPβ mutant binding. Nevertheless, the extensive decoration with PTMs in conjunction with reprogramming data provided here suggest that C/EBPβ integrates extracellular signals to accomplish alternative differentiation into diverse cells of the innate immune system.

## Materials and Methods

### Ethics Statement

All mice were bred and maintained in accordance with guidelines from institutional Animal Care Committee under specific pathogen-free animal facilities at the MDC/Charité. Experiments were approved by the Commission for Animal Experiments at the MDC and the Berlin Office of Health (LAGeSo), Permit Number T 0339/08. For isolation of cells, mice were sacrificed by euthanasia using carbon dioxide inhalation followed by cervical dislocation. All efforts were made to minimize animal suffering.

### Mouse Strains, Cell Sorting and FACS Analyses

Primary B cell progenitors were obtained from bone marrow of 3–6 months old C57BL/6 or *C/EBPβ^−/−^* mice [48]. After erythrolysis, cells were incubated with non-B cell lineage (Lin) biotin-coupled antibodies against Gr-1 (RB6-8C5), CD11b (M1/70), CD4 (GK1.5), CD8 (53-6.7), TER-119 (TER-119), and CD49b (DX5) (Biolegend) and Lin^+^ cells were depleted using Dynabeads sheep anti-Rat IgG (Invitrogen). Cells were then stained with B220-PE Cy7 (RA3-6B2), CD19-FITC (6D5), Gr-1-PE (RB6-8C5), SA-APC Cy7 (Biolegend), IgM-APC (II/41) (BD Pharmingen), and DAPI and Lin^–^ B220^+^ IgM^–^ CD19^+/−^ pre-pro/pro/pre B cells were sorted by FACS.

For the FACS analyses, after Fc blocking with rat anti-mouse CD16/32 antibody (BD Pharmingen) cells were stained with the following antibodies: rat anti-mouse CD11b-PerCP Cy5.5 (M1/70), CD11b-APC Cy7 (M1/70), CD11b-PE (M1/70), CD19-APC (6D5), CD19-PE Cy7 (6D5), CD45-PE Cy7 (30-F11), Gr-1-APC Cy7 (RB6-8C5), Ly-6C-APC Cy7 (HK1.4), Ly-6G-APC (1A8), CD115-PE (M-CSFR, Cl. AFS98), CD115-APC (M-CSFR, Cl. AFS98), F4/80-Pacific blue (A3-1), MHC-II-PE (I-A/I-E, Cl. M5/114.15.2) (all from Biolegend), CD86-PE (B7-2), hamster anti-mouse CD11c-APC (HL3) and CD11c-V450 (HL3) (BD Pharmingen). 7-AAD (BD Pharmingen) or DAPI (Invitrogen, Molecular probes) were added to discriminate cell viability. Samples were run on FACS Canto machine (BD Biosciences, BD Diva Software) and analyzed with FlowJo software.

### Retroviral Vectors, Infection and Cell Culture

The C/EBPβ (GI:148539989) LAP* start site was optimized regarding the Kozak consensus sequence and CR-deletion mutants were published before [14]. C/EBPβ point mutations were obtained by site directed mutagenesis using the QuikChange site-directed mutagenesis kit (Stratagene). All C/EBP constructs were cloned into the MIEG3 (MSCV-IRES-EGFP) retroviral vector. Purified B cell progenitors were seeded at 2×10^5^ cells/ml in IMDM medium with 20% hiFCS, 50 µM 2-mercaptoethanol (Invitrogen) and 10 ng/ml IL-7, SCF, Flt-3L, and infected with viral supernatant plus polybrene (Sigma; 8 µg/ml) [10]. Infected cells were transferred into HTS Transwell-24 well (Corning) supplemented with 10 ng/ml IL-7, SCF, Flt-3L, IL-3 and M-CSF (Peprotech) and co-cultured with S17 cells [49] pretreated with 10 µg/ml mitomycin. Expression of C/EBPβ constructs was determined by GFP cytofluorometric read-out, correct protein sizes were assessed by immunobloting, and intracellular protein staining confirmed expression of C/EBPβ proteins in the retrovirally infected primary B cell progenitors (Fig. S1C, D).

### Cytospins

GFP^+^ CD11b^+^ and GFP^+^ CD19^+^ cells were sorted by FACS 9 days after retroviral infection and cytospins were performed. Slides were fixed in 100% methanol and stained with May-Grunwald and Giemsa (Sigma).

### RNA Extraction and mRNA Expression Analysis by Nanostring Technology

Total RNA was extracted from *C/EBPβ^−/−^* B cell progenitors 6 days after infection with C/EBPβ constructs and sorting of the CD11b^+^ reprogrammed cells or from bone marrow-derived macrophages (control, 6 days *in vitro* cultured) using RNeasy Micro Kit (QIAGEN) according to the manufacture’s recommendations. mRNA counts were determined using Nanostring technology [50] after background subtraction and normalization to three house-keeping genes (*Gapdh*, *Tbp*, *Ppia*). Expression below the background level was set to value “1”. After log_2_ transformation, data were subjected to hierarchical clustering using Euclidean Distance to generate a gene and sample tree (MeV software).

### Statistical Analysis

In all experiments, data are presented as mean ± SEM (standard error of the mean). Statistical analyses were done on Prism 4.0a (GraphPad Software) applying unpaired two-tailed t test for the calculation of the P-value. The statistical significance of the P-value was defined as: P>0.05 - not significant, P = 0.01–0.05 - significant (*), P = 0.001–0.01 - very significant (**), P<0.001 - extremely significant (***).

More Materials and Methods could be found in the **Materials and Methods S1**.

## Supporting Information

Figure S1 **FACS sorting strategy, rearrangements in IgH gene loci and C/EBPβ expression in the C/EBPβ reprogrammed myeloid cells (related to** **Figure 1** **).** A. Bone marrow single cell suspension was prepared and cells stained, as described in Materials and Methods. Lin^–^ B220^+^ IgM^–^ CD19^+/−^ pre-pro/pro/pre B cell progenitors were sorted for the reprogramming experiments. Lin^+^ cells were cultured *in vitro* for obtaining bone marrow-derived macrophages (MPh) for negative controls for IgH rearrangement PCR. Lin^–^ B220^+^ IgM^+^ bone marrow immature B cells and spleenic B220^+^ B cells were sorted for positive rearrangement PCR controls. B. PCR for D-J rearrangements in IgH locus. CD11b^+^ reprogrammed myeloid cells and CD19^+^ MSCV-, LIP- and ΔCR3,4-infected B cells were sorted and PCR for D-J rearrangements in the IgH locus was performed. Controls: WT bone marrow-derived macrophages (MPh) and spleenic B cells. Data shown are representative from multiple experiments. C. Protein expression of the C/EBPβ WT and deletion constructs in the virus-packaging cell line PlatE. The size of the proteins is according to the size of the deletions. D. Intracellular C/EBPβ protein staining in the reprogrammed cells. The relative C/EBPβ expression in the virus-infected cells was calculated as described in Materials and Methods S1. The endogenous C/EBPβ expression level in WT bone marrow-derived macrophages (MPh) was also assessed. The relative C/EBPβ expression values varied between the different experiments, however the tendencies were highly reproducible.
(TIF)

Figure S2 **Reprogramming of WT and** ***C/EBPβ^−/−^*** **B cell progenitors by C/EBPα and** **C/EBPβ (related to** **Figure 1** **).** A. Representative FACS profiles of the C/EBPβ infected WT B cell progenitors at 6 and 9 dpi. FACS plots represent GFP^+^ gated cell population, B cells - control uninfected GFP^–^ B cell progenitors. Similar outcomes were obtained from at least two repeat experiments. B. Percentage of *C/EBPβ^−/−^* B cell progenitors infected with C/EBPβ WT and mutants expressing the B cell marker CD19 or the myeloid marker CD11b at 6 dpi. Intermediates (CD19^+^ CD11^+^ cells) are also included. Graphs represent GFP^+^ gated cell population, B cells - control uninfected GFP^–^ B cell progenitors. Values represent mean ± SEM from two and more repeat experiments. C. Percentage of WT and *C/EBPβ^−/−^* B cell progenitors infected with WT C/EBPα p42 and p30 expressing the B cell marker CD19 or the myeloid marker CD11b at 6 dpi. Intermediates (CD19^+^ CD11^+^ cells) are also included. Graphs represent GFP^+^ gated cell population. Values for *C/EBPβ^−/−^* B cell progenitors represent mean ± SEM from three repeat experiments.
(TIF)

Figure S3 **Heterogeneity among reprogrammed myeloid cells and lack of differential apoptosis between the subpopulations of reprogrammed cells (related to** **Figure 3** **).** A. Phagocytosis assay was performed after 10 days *in vitro* reprogramming. Red line represents cells incubated with fluorescent latex beads and the black line - the auto-fluorescence of the untreated samples. For MSCV-infected cells histograms represent GFP^+^ CD19^+^ population, whereas C/EBPβ-infected reprogrammed cells were gated on GFP^+^ CD11b^+^ cells. As positive controls for phagocytic capacity, bone marrow-derived macrophages (MPh) were used. Similar outcomes were obtained in two or more repeat experiments. B. Apoptosis assay based on AnnexinV staining and evaluated by FACS. Dead cells were excluded by DAPI staining and the apoptosis assessment was done after gating on the different GFP^+^ cell populations (CD19^+^, CD11b^+^ Gr-1^–^ and CD11b^+^ Gr-1^+^). na – no available cells with these surface characteristics. The graph represents data from four independent experiments. C. Expression of Ly-6C and M-CSFR myeloid cell markers on the reprogrammed cells at 6 and 9 dpi. FACS plots represent GFP^+^ CD11b^+^ cell population. For MSCV-infected cells FACS plots represent GFP^+^ CD19^+^ cells. The myeloid cell marker staining was repeated in at least two independent experiments and similar results were obtained.
(TIF)

Table S1 **C/EBPβ WT and mutant constructs display different B-to-myeloid cell reprogramming kinetics (related to** **Figure 1** **).**
(DOC)

Table S2 **Differential Ly-6C expression on CD11b^+^ cells reprogrammed by WT and mutant C/EBPβ (related to** **Figure 3** **).**
(DOC)

Materials and Methods S1 **Supplementary Materials and Methods**
(DOC)
