# Supplementary figures and images for: Lymphoid to Myeloid Cell Trans-Differentiation Is Determined by C/EBPβ Structure and Post-Translational Modifications
Source: PLoS One. 2013 Jun 5;8(6):e65169. doi: 10.1371/journal.pone.0065169 (PMC3674013; doi:10.1371/journal.pone.0065169)

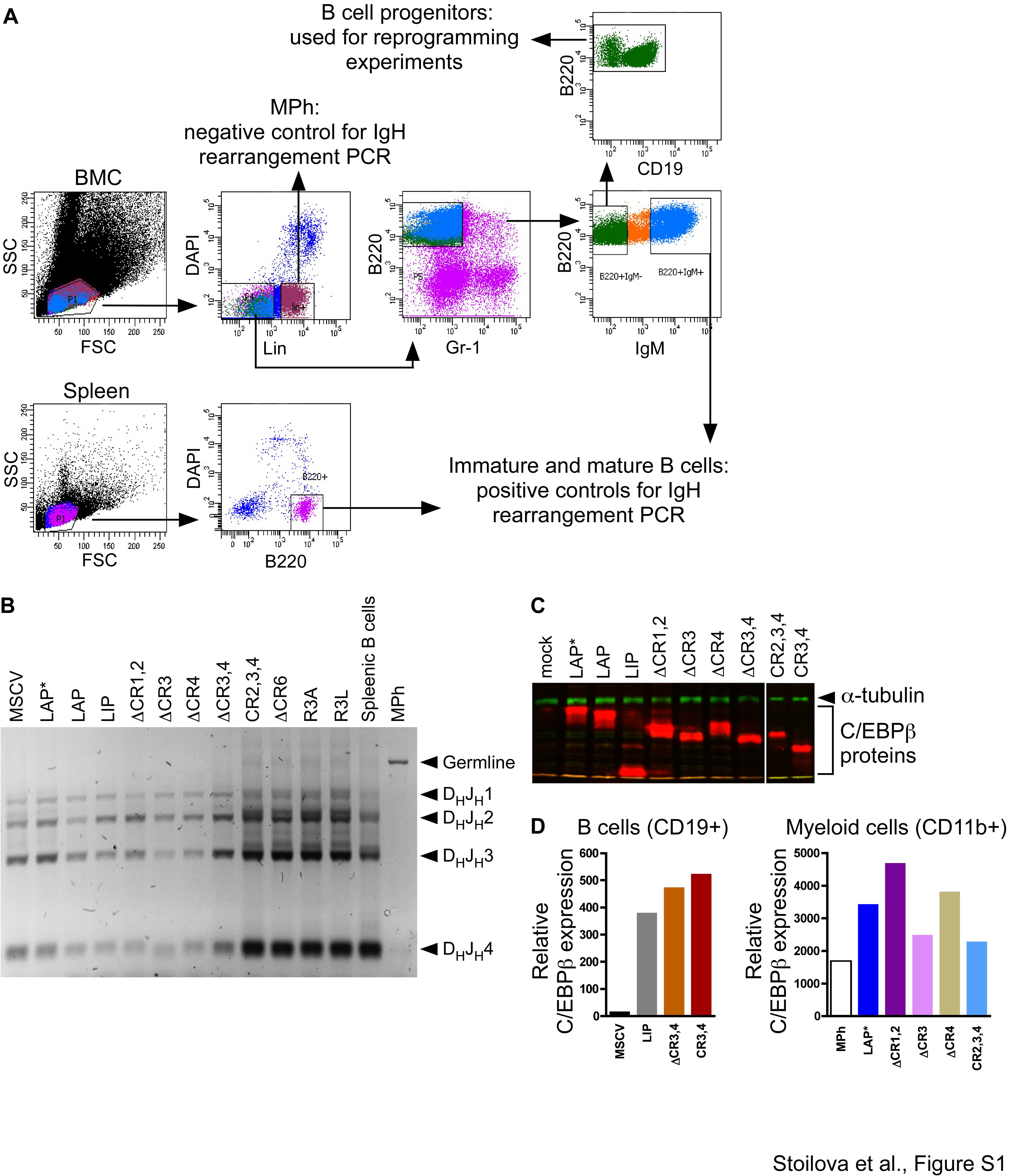

Supplement: Figure S1 — FACS sorting strategy, rearrangements in IgH gene loci and C/EBPβ expression in the C/EBPβ reprogrammed myeloid cells (related to Figure 1 ). A. Bone marrow single cell suspension was prepared and cells stained, as described in Materials and Methods. Lin– B220+ IgM– CD19+/− pre-pro/pro/pre B cell progenitors were sorted for the reprogramming experiments. Lin+ cells were cultured in vitro for obtaining bone marrow-derived macrophages (MPh) for negative controls for IgH rearrangement PCR. Lin– B220+ IgM+ bone marrow immature B cells and spleenic B220+ B cells were sorted for positive rearrangement PCR controls. B. PCR for D-J rearrangements in IgH locus. CD11b+ reprogrammed myeloid cells and CD19+ MSCV-, LIP- and ΔCR3,4-infected B cells were sorted and PCR for D-J rearrangements in the IgH locus was performed. Controls: WT bone marrow-derived macrophages (MPh) and spleenic B cells. Data shown are representative from multiple experiments. C. Protein expression of the C/EBPβ WT and deletion constructs in the virus-packaging cell line PlatE. The size of the proteins is according to the size of the deletions. D. Intracellular C/EBPβ protein staining in the reprogrammed cells. The relative C/EBPβ expression in the virus-infected cells was calculated as described in Materials and Methods S1. The endogenous C/EBPβ expression level in WT bone marrow-derived macrophages (MPh) was also assessed. The relative C/EBPβ expression values varied between the different experiments, however the tendencies were highly reproducible. (TIF) [file pone.0065169.s001.tif]

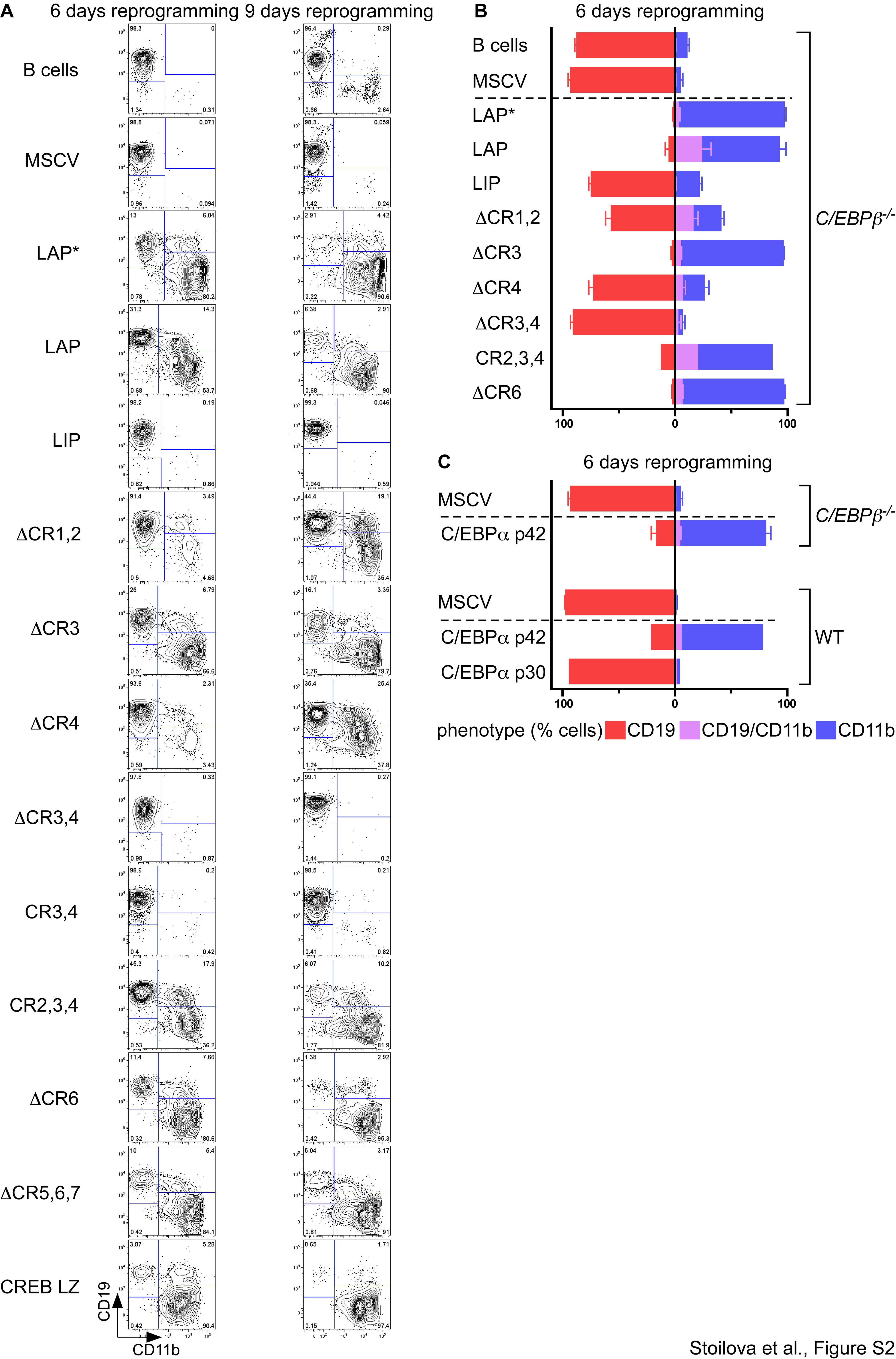

Supplement: Figure S2 — Reprogramming of WT and C/EBPβ−/− B cell progenitors by C/EBPα and C/EBPβ (related to Figure 1 ). A. Representative FACS profiles of the C/EBPβ infected WT B cell progenitors at 6 and 9 dpi. FACS plots represent GFP+ gated cell population, B cells - control uninfected GFP– B cell progenitors. Similar outcomes were obtained from at least two repeat experiments. B. Percentage of C/EBPβ−/− B cell progenitors infected with C/EBPβ WT and mutants expressing the B cell marker CD19 or the myeloid marker CD11b at 6 dpi. Intermediates (CD19+ CD11+ cells) are also included. Graphs represent GFP+ gated cell population, B cells - control uninfected GFP– B cell progenitors. Values represent mean ± SEM from two and more repeat experiments. C. Percentage of WT and C/EBPβ−/− B cell progenitors infected with WT C/EBPα p42 and p30 expressing the B cell marker CD19 or the myeloid marker CD11b at 6 dpi. Intermediates (CD19+ CD11+ cells) are also included. Graphs represent GFP+ gated cell population. Values for C/EBPβ−/− B cell progenitors represent mean ± SEM from three repeat experiments. (TIF) [file pone.0065169.s002.tif]

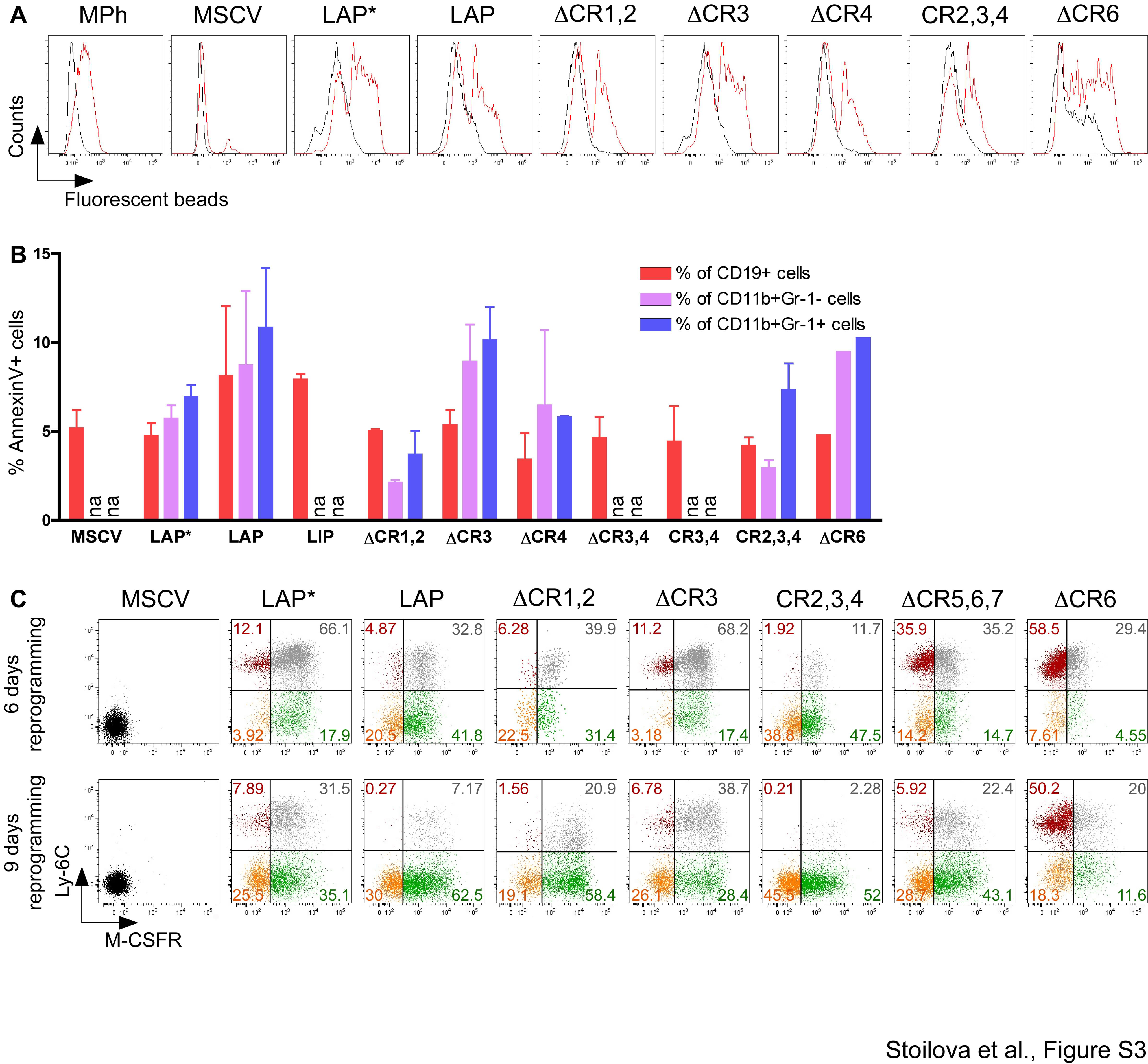

Supplement: Figure S3 — Heterogeneity among reprogrammed myeloid cells and lack of differential apoptosis between the subpopulations of reprogrammed cells (related to Figure 3 ). A. Phagocytosis assay was performed after 10 days in vitro reprogramming. Red line represents cells incubated with fluorescent latex beads and the black line - the auto-fluorescence of the untreated samples. For MSCV-infected cells histograms represent GFP+ CD19+ population, whereas C/EBPβ-infected reprogrammed cells were gated on GFP+ CD11b+ cells. As positive controls for phagocytic capacity, bone marrow-derived macrophages (MPh) were used. Similar outcomes were obtained in two or more repeat experiments. B. Apoptosis assay based on AnnexinV staining and evaluated by FACS. Dead cells were excluded by DAPI staining and the apoptosis assessment was done after gating on the different GFP+ cell populations (CD19+, CD11b+ Gr-1– and CD11b+ Gr-1+). na – no available cells with these surface characteristics. The graph represents data from four independent experiments. C. Expression of Ly-6C and M-CSFR myeloid cell markers on the reprogrammed cells at 6 and 9 dpi. FACS plots represent GFP+ CD11b+ cell population. For MSCV-infected cells FACS plots represent GFP+ CD19+ cells. The myeloid cell marker staining was repeated in at least two independent experiments and similar results were obtained. (TIF) [file pone.0065169.s003.tif]
