# Supplementary material for: Lymphoid to Myeloid Cell Trans-Differentiation Is Determined by C/EBPβ Structure and Post-Translational Modifications
Source: PLoS One. 2013 Jun 5;8(6):e65169. doi: 10.1371/journal.pone.0065169 (PMC3674013; doi:10.1371/journal.pone.0065169)
Supplement: Table S1 — C/EBPβ WT and mutant constructs display different B-to-myeloid cell reprogramming kinetics (related to Figure 1 ). (DOC) [file pone.0065169.s004.doc]

**Table S1. C/EBPβ WT and mutant constructs display different B-to-myeloid cell reprogramming kinetics (related to Figure 1).**

|  | 6 days reprogramming | | | | 9 days reprogramming | | | |
| --- | --- | --- | --- | --- | --- | --- | --- | --- |
|  | N ^a)^ | % CD11b^+^ cells ^b)^ | P value ^c)^ | Signifi-  cance ^d)^ | N ^a)^ | % CD11b^+^ cells ^b)^ | P value ^c)^ | Signifi-  cance ^d)^ |
| B cells | 11 | 4.3 ± 0.73 | <0.0001 | *** | 11 | 5.5 ± 1.0 | <0.0001 | *** |
| MSCV | 15 | 1.4 ± 0.3 | <0.0001 | *** | 12 | 1.8 ± 0.6 | <0.0001 | *** |
| LAP* | 15 | 85.4 ± 2.7 | reference | - | 14 | 92.6 ± 1.7 | reference | - |
| LAP | 8 | 70.1 ± 6.7 | 0.020 | * | 6 | 92.8 ± 1.4 | 0.948 | ns |
| LIP | 5 | 1.6 ± 0.8 | <0.0001 | *** | 3 | 1.8 ± 0.8 | <0.0001 | *** |
| ΔCR1,2 | 5 | 11.5 ± 6.0 | <0.0001 | *** | 5 | 44.7 ± 12.8 | <0.0001 | *** |
| ΔCR3 | 5 | 75.5 ± 3.8 | 0.072 | ns | 5 | 88.5 ± 3.1 | 0.237 | ns |
| ΔCR4 | 4 | 8.9 ± 3.4 | <0.0001 | *** | 4 | 61.6 ± 11.0 | 0.0002 | *** |
| ΔCR3,4 | 5 | 1.7 ± 0.9 | <0.0001 | *** | 5 | 2.1 ± 1.0 | <0.0001 | *** |
| CR3,4 | 2 | 0.5 ± 0.1 | <0.0001 | *** | 2 | 1.0 ± 0.1 | <0.0001 | *** |
| CR2,3,4 | 6 | 66.8 ± 6.2 | 0.004 | ** | 6 | 93.6 ± 0.8 | 0.708 | ns |
| ΔCR6 | 6 | 91.1 ± 2.0 | 0.217 | ns | 5 | 97.0 ± 0.8 | 0.150 | ns |
| ΔCR5,6,7 | 4 | 79.2 ± 7.6 | 0.347 | ns | 5 | 92.6 ± 2.6 | 0.992 | ns |
| CREB LZ | 4 | 95.6 ± 1.2 | 0.071 | ns | 3 | 97.1 ± 1.0 | 0.2510 | ns |

^a)^ N - number of experiments.

^b)^ Percentage of cells infected with WT and mutant C/EBPβ retroviral constructs expressing CD11b surface antigen (CD19^+/–^ CD11b^+^ cells) 6 or 9 days after the infection. B cells - control uninfected GFP^–^ B cell progenitors. Values represent mean ± SEM from a different number of experiments (N).

^c)^ P values were calculated versus LAP* at 6 or 9 dpi.

^d)^ Significance was defined as described in Materials and Methods.
