# Supplementary material for: Lymphoid to Myeloid Cell Trans-Differentiation Is Determined by C/EBPβ Structure and Post-Translational Modifications
Source: PLoS One. 2013 Jun 5;8(6):e65169. doi: 10.1371/journal.pone.0065169 (PMC3674013; doi:10.1371/journal.pone.0065169)
Supplement: Table S2 — Differential Ly-6C expression on CD11b+ cells reprogrammed by WT and mutant C/EBPβ (related to Figure 3 ). (DOC) [file pone.0065169.s005.doc]

**Table S2. Differential Ly-6C expression on CD11b^+^ cells reprogrammed by WT and mutant C/EBPβ (related to Figure 3).**

|  | LAP* | LAP | ΔCR1,2 | ΔCR3 | ΔCR4 | CR2,3,4 | ΔCR6 |
| --- | --- | --- | --- | --- | --- | --- | --- |
| 6 dpi ^a)^ | 77 ± 1.7 | 39 ± 8.1 | 53 ± 6.9 | 78 ± 1.5 | 69 ± 1.8 | 18 ± 1.8 | 85 ± 1.5 |
| 9 dpi ^a)^ | 51 ± 3.2 | 13 ± 2.4 | 19 ± 3.4 | 47 ± 1.1 | 39 ± 0.56 | 5.7 ± 2.0 | 71 ± 5.5 |
| N ^b)^ | 7 | 4 | 2 | 2 | 2 | 5 | 4 |
| P value ^c)^ | <0.0001 | 0.021 | 0.047 | 0.003 | 0.004 | 0.002 | 0.046 |
| Significance ^d)^ | Yes (***) | Yes (*) | Yes (*) | Yes (**) | Yes (**) | Yes (**) | Yes (*) |

^a)^ Percentage of cells infected with WT and mutant C/EBPβ retroviral constructs expressing Ly-6C surface antigen 6 or 9 days after the infection. The total percentage of GFP^+^ CD11b^+^ cells was set to 100%. Values represent mean ± SEM.

^b)^ N - number of experiments.

^c)^ P values were calculated by unpaired t test 6 versus 9 days reprogramming.

^d)^ Significance was defined as described in Materials and Methods.
