# Supplementary material for: Lymphoid to Myeloid Cell Trans-Differentiation Is Determined by C/EBPβ Structure and Post-Translational Modifications
Source: PLoS One. 2013 Jun 5;8(6):e65169. doi: 10.1371/journal.pone.0065169 (PMC3674013; doi:10.1371/journal.pone.0065169)
Supplement: Materials and Methods S1 — Supplementary Materials and Methods (DOC) [file pone.0065169.s006.doc]

**Supplementary Materials and Methods**

**Cell sorting**

Spleenic cells were Fc blocked with rat anti-mouse CD16/32 antibody (BD Pharmingen), stained for the FACS sorting with B220-FITC (RA3-6B2) (Biolegend) and DAPI and B220^+^ DAPI^–^ cells were sorted.

**Retroviral vectors and retrovirus production**

All C/EBPβ mutations were validated by sequencing. Retroviral stocks were prepared by transfection of Plat-E cells using CaCl_2_ standard protocol.

**Cell culture**

For obtaining bone marrow-derived macrophages, FACS sorted Lin^+^ bone marrow cells were cultured in IMDM medium, 20% hiFCS, 50 μM 2-mercaptoethanol (Invitrogen) supplemented with 10 ng/ml M-CSF (Peprotech).

**Intracellular protein staining, AnnexinV staining and Phagocytosis assay**

For the intracellular C/EBPβ staining, after extracellular marker staining, fixation and permeabilization with BD Cytofix/Cytoperm Buffer, cells were stained with anti-C/EBPβ rabbit antibody (Santa Cruz, C-19) and then with anti – rabbit PE conjugated antibody (Jackson Immuno Research). Antibody dilutions and washing steps were performed in 1xPerm Wash Buffer (BD). The relative C/EBPβ expression in the virus-infected cells was calculated as the change (mean) of C/EBPβ fluorescence of the probes incubated with or without the primary C/EBPβ antibody and after subtraction of the endogenous expression for each sample. For AnnexinV staining, 6 days after the infection, cells were incubated with Mouse BD Fc Block (BD Pharmingen), stained for surface marker expression, and then with AnnexinV-APC (BD Pharmingen) and DAPI in 1x AnnexinV binding buffer (Biolegend) according to manufacture’s instructions. For the phagocytosis assays, 10 days reprogrammed cells were incubated with 1,0 μm Blue (365/415) fluorescent carboxylate-modified microspheres (Molecular Probes) and fixed with 1% paraformaldehyde before FACS analyses.

**Western Blotting**

Before the Western blotting, cells were lysed on ice in RIPA buffer (50mM Tris/HCL, pH 7.4, 150mM NaCl, 1% NP–40, 0,25% Sodium Deoxycholat, 0,1% SDS, 1mM EDTA) supplemented with protease inhibitors (1 mM Na_3_VO_4_, phenanthroline, DTT and PMSF; 10 μg/ml pepstatin, aprotenin A and leupeptin (all AppliChem)). Proteins were separated by SDS-PAGE, followed by blotting and detection with polyclonal rabbit anti-C/EBPβ (Santa Cruz, C-19 or Leutz lab) and monoclonal mouse anti-α-tubulin antibody (Santa Cruz). Blots were incubated with IRDye™ 680 goat anti-rabbit and IRDye™ 800CW goat anti-mouse IgG (LI-COR) and scanned with the Odyssey Infrared Imaging System (LI-COR).

**Immunoglobulin Gene Rearrangements**

GFP^+^ CD11b^+^ and GFP^+^ CD19^+^ cells from S17 co-cultures were sorted by FACS at 6 or 9 dpi. After extraction of genomic DNA, D_H_J_H_ rearrangements were examined by the primer pair D_FS_ and J_H_4A together with D_Q_52 for germ line locus configuration . Bone marrow-derived macrophages cultured *in vitro* and splenic B cells were used as controls.

**Supplementary References**

1. Ehlich A, Martin V, Muller W, Rajewsky K (1994) Analysis of the B-cell progenitor compartment at the level of single cells. Current biology : CB 4: 573-583.
